# CyTargetLinker app update: A flexible solution for network extension in Cytoscape

**DOI:** 10.12688/f1000research.14613.2

**Published:** 2019-08-13

**Authors:** Martina Kutmon, Friederike Ehrhart, Egon L. Willighagen, Chris T. Evelo, Susan L. Coort

**Affiliations:** 1 Department of Bioinformatics - BiGCaT, NUTRIM, Maastricht University, Maastricht, 6229 ER, The Netherlands; 2 Maastricht Centre for Systems Biology (MaCSBio), Maastricht University, Maastricht, 6229 ER, The Netherlands; 3 GKC-Rett Expertise Centre, Maastricht University Medical Center, Maastricht, 6200 MD, The Netherlands

**Keywords:** Cytoscape, CyTargetLinker, network extension, network visualization, regulatory networks, data integration

## Abstract

Here, we present an update of the open-source CyTargetLinker app for Cytoscape ( http://apps.cytoscape.org/apps/cytargetlinker) that introduces new automation features. CyTargetLinker provides a simple interface to extend networks with links to relevant data and/or knowledge extracted from so-called linksets. The linksets are provided on the CyTargetLinker website ( https://cytargetlinker.github.io/) or can be custom-made for specific use cases. The new automation feature enables users to programmatically execute the app’s functionality in Cytoscape (command line tool) and with external tools (e.g. R, Jupyter, Python, etc). This allows users to share their analysis workflows and therefore increase repeatability and reproducibility. Three use cases demonstrate automated workflows, combinations with other Cytoscape apps and core Cytoscape functionality. We first extend a protein-protein interaction network created with the stringApp, with compound-target interactions and disease-gene annotations. In the second use case, we created a workflow to load differentially expressed genes from an experimental dataset and extend it with gene-pathway associations. Lastly, we chose an example outside the biological domain and used CyTargetLinker to create an author-article-journal network for the five authors of this manuscript using a two-step extension mechanism.

With 400 downloads per month in the last year and nearly 20,000 downloads in total, CyTargetLinker shows the adoption and relevance of the app in the field of network biology. In August 2019, the original publication was cited in 83 articles demonstrating the applicability in biomedical research.

## Introduction

The CyTargetLinker app provides a flexible and simple way to extend networks in Cytoscape ^1^ with links to (prior) knowledge from external sources. Since its first release in 2013 ^2^, CyTargetLinker has been downloaded more than 19,000 times and used in numerous studies. These applications in biological studies range from the creation of a microRNA-gene association network for lipid diseases ^3^ or Alzheimer’s disease ^4^ to the application of algorithms for drug sensitivity prediction ^5^.

While the app was originally intended to be used for the extension of biological networks with regulatory interactions, researchers have used CyTargetLinker to integrate knowledge about many different types of relationships (e.g. pathway associations and disease annotations). Therefore, we renamed the previously used *Regulatory Interaction Networks* (RegINs) to *linksets* to make the broader applicability more explicit. Moreover, the generation of linksets, either manually or in an automated manner, has become more user-friendly.

In this new version of CyTargetLinker, we introduce an automation feature that allows programmatic access to the app functionality. In the Results section, we present three use cases that highlight the app’s purpose, how it can be easily combined with other Cytoscape apps and the advantages of the automation. Whereas the first two use cases have a biological nature, the third use case demonstrates the broader applicability with a non-biological example. Additionally, the website and tutorials have been updated and restructured ( https://cytargetlinker.github.io/).

## Methods

The newest version of CyTargetLinker (4.0.0+) was developed for Cytoscape (3.6.0+) which introduces a new interface for automation that can make apps callable as services by the Cytoscape Command scripts, Python and R. This promotes open and reproducible data analysis, and simple integration with other apps. CyTargetLinker can be installed through the Cytoscape app store.

### LinkSets

On the CyTargetLinker website, we provide a variety of linksets for regulatory interactions, pathway associations and disease annotations ( https://cytargetlinker.github.io/pages/linksets). Additionally, we deliver a simple Java program to convert tab delimited text files into XGMML linksets that can be used with CyTargetLinker ( https://github.com/CyTargetLinker/linksetCreator). Using BridgeDb ^6^, a framework for finding and mapping database identifiers, the script enables the support of multiple identifier systems for biological entities.

### Application programming interface

While CyTargetLinker can still be used through the Cytoscape graphical user interface (see online tutorials), we would like to highlight the novel application programming interface (API) that allows the programmatic execution of the app’s functionality.

CyTargetLinker provides a set of API methods to automise the extension of networks ( Table 1). The key function is the “extend” function, which parses the provided linksets and extracts relevant interactions for the selected network. The user can then choose to use the CyTargetLinker visual style and the force-directed layout. Often, users want to integrate knowledge for the same interaction type from different resources. With the “filterOverlap” function, users can visualise only those interactions that are supported by multiple resources.

**Table 1.** CyTargetLinker API. List of the API methods of CyTargetLinker, their parameters and a general description.

API Method	Parameters	Task
**version**		Returns the version of the CyTargetLinker app
**extend**	direction, idAttribute, linkSetDirectory, linkSetFiles, network	Extend network with interactions from linksets
**applyVisualstyle**	network	Apply the CyTargetLinker visual style to the extended network
**applyLayout**	network	Apply a force-directed layout
**filterOverlap**	network, overlap	Filter extension and show only links supported by *n* linksets
**showPanel**	show	Show the CyTargetLinker result panel

## Use cases

The broad applicability of CyTargetLinker will be demonstrated in three different use cases. The focus lies on the automation of the analysis and the R scripts for each use case are provided in the automation repository on GitHub. We chose to present two biological and one non-biological use cases to demonstrate the flexibility of the app.

### Use cases highlighting the new automation functionality

**Figure 1.**
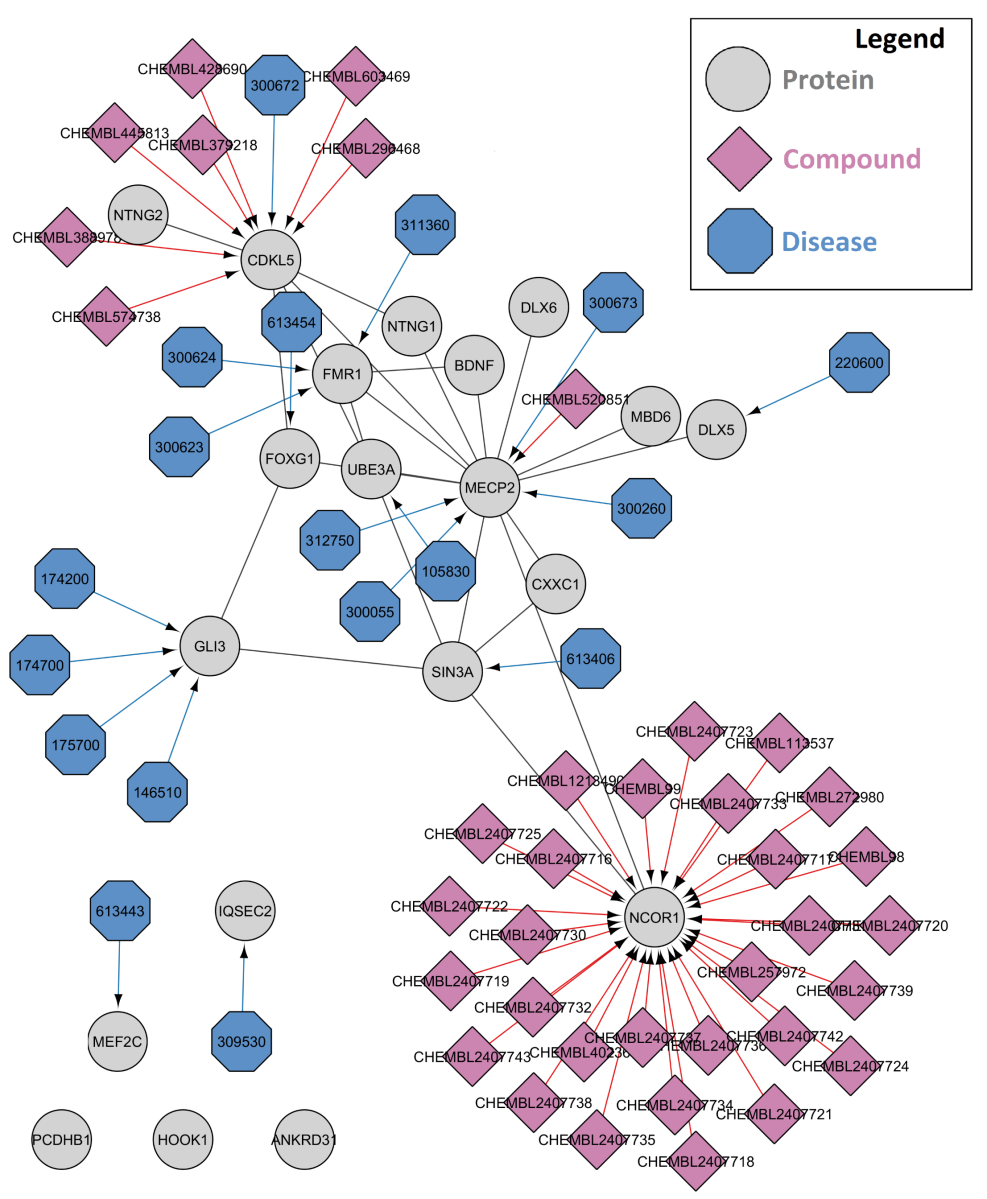
Compound-gene-disease network for Rett Syndrome. The protein-protein interaction network for Rett syndrome was created using the disease query option of the stringApp for Cytoscape. The proteins are represented as gray circles. Then, CyTargetLinker was used to extend the network with compounds from ChEMBL (purple diamonds) and disease annotations from OMIM (blue octagons).

***Use case 1: Investigating drug-targets and disease associations for a Rett syndrome protein-protein interaction network.*** Rett syndrome is a rare disease caused by a mutation in the methyl-CpG-binding protein 2 (MECP2) gene ^7^. In this use case, we used the stringApp ^8^ of Cytoscape to create a protein-protein interaction (PPI) network for Rett syndrome (Disease Query). The PPI network is then extended using CyTargetLinker with compound-target interactions from ChEMBL ^9, 10^ and disease-gene associations from a manually curated subset for rare diseases from OMIM ^11^. ChEMBL is an open online bioactivity database containing information about compounds, their bioactivity and their possible targets (including proteins). OMIM is a comprehensive collection of human genetic phenotypes and their associated human genes. First, the stringApp was used to create a Rett syndrome PPI (query=“Rett syndrome”, cutoff=0.4, limit=20). Using CyTargetLinker, the network was extended with 37 compound-target interactions from ChEMBL and 18 gene-disease associations from OMIM (see Figure 1).

The following API command was used to extend the network with compound-target and disease-gene information:


cytargetlinker extend
idAttribute=”display name”
linkSetFiles=”../LinkSets/chembl_23_hsa_20180126.xgmml,
../LinkSets/omim-rare-disease-has-20180411.xgmml”
network=current


***Use case 2: Pathway associations for differentially expressed genes in Rett syndrome.*** For this use case, we selected a list of differentially expressed genes in the Purkinje cells located in the cerebellar cortex of the brain of a Mecp2 ^*−/y*^ mouse model ^12, 13^ for Rett syndrome. Next, we investigated in which biological processes these altered genes are involved. Using the pathway annotations from the WikiPathways database ^14^, CyTargetLinker adds the pathway information and creates a pathway-gene network.

**Figure 2.**
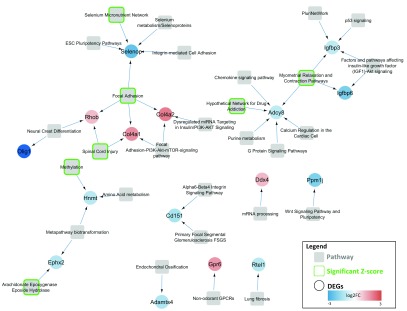
Gene-Pathway network for differentially expressed genes (DEGs) in Purkinje cells of Mecp2 ^−/ *y*^ vs. wild type. DEGs in a mouse model for Rett syndrome (Mecp2 ^−/ *y*^ mice) were selected and imported in Cytoscape (circular nodes). The genes are colored based on changes in gene expression (blue=down-regulated, red=up-regulated). Thereafter, gene-pathway associations from WikiPathways were added and the pathways are shown as gray rectangles. Genes without pathway annotations have been removed. The green border color indicates pathways that are significantly altered based on over-representation analysis in PathVisio (Z-Score > 1.96).

From the dataset, we extracted 65 genes with an absolute log2 fold change larger than 1. Only 16 genes are present in one or more pathways of the curated mouse pathway collection from WikiPathways. Figure 2 shows the resulting gene-pathway network. Genes without pathway annotations have been removed. Differential gene expression is shown on the gene nodes (blue = down, red = up) and green border color of the pathway nodes indicates that the pathway has been identified as significantly affected through overrepresentation analysis in the pathway analysis tool PathVisio ^15^.

The following API command was used to extend the network with pathway information:


cytargetlinker extend
idAttribute=”shared name”
linkSetFiles=”../LinkSets/wikipathways-mm-20180410.xgmml”
network=current
direction=SOURCES


**Figure 3.**
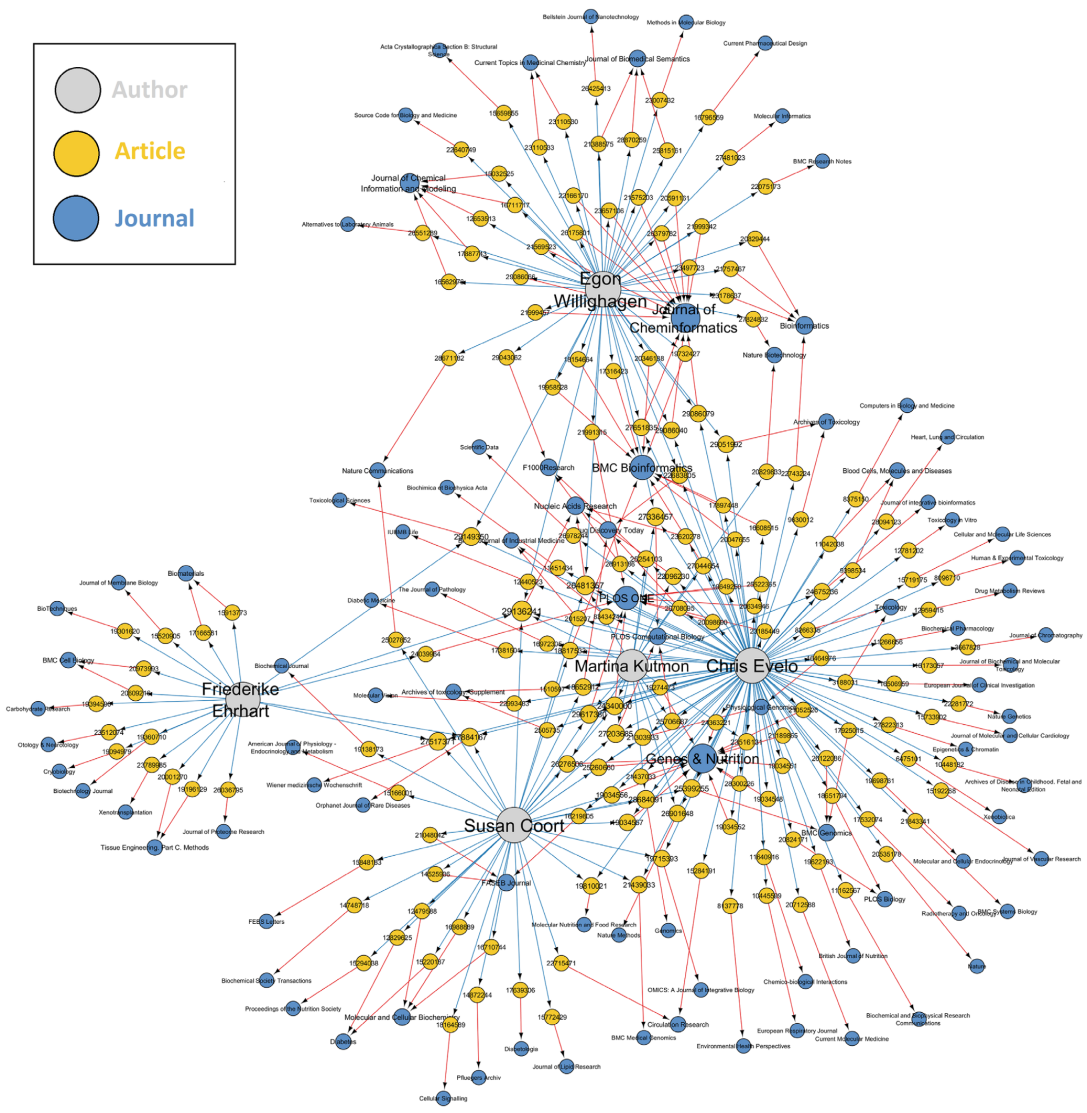
Author-Article-Journal network. For the five authors of this manuscript, two custom linksets were used to first add author-article relationships and in a second step add article-journal relationships.

***Use case 3: Author-publication-journal network.*** This example uses two custom made linksets for author-article and article-journal relationships from Wikidata ^16– 18^. After loading the initial five author nodes in Cytoscape, we performed a two-step extension with CyTargetLinker. We first added publications from the author-article linkset and then the journals from the article-journal linkset, see Figure 3. Author nodes are colored in gray, articles in yellow and journals in green. The network clearly shows the collaborations and diversity between the authors. Layout and visual style was slightly adapted manually in the graphical user interface to improve the readability of the network.

The following API commands were used to extend the network first with publication and then with journal information:


cytargetlinker extend
idAttribute=”shared name”
linkSetFiles=”../LinkSets/publications.xgmml”
network=current

cytargetlinker extend
idAttribute=”shared name”
linkSetFiles=”../LinkSets/journals.xgmml”
network=current


## Discussion

One of the major challenges in science is the reproducibility of results presented in articles. Besides the challenges in reproducibility of experiments, the computational analyses are also often unclear and insufficiently described ^19^. Automation of analysis workflows enables researchers to share the details of their computational analyses and enables simple reproducibility of the results.

Here, we introduce the new version of the CyTargetLinker app, which provides full programmatic execution of the functions from within Cytoscape (command line tool) and external tools (e.g. R, Jupyter, Python, etc). The network extension is therefore reproducible and repeatable with other input data. Consequently, users can build scripts that run common analysis workflows and combine CyTargetLinker with other apps, as shown in Use case 1 (stringApp). The integration of CyTargetLinker in Cytoscape gives access to a powerful set of visualization options, as demonstrated in use case 2.

As part of the Cytoscape tutorials collection for online presentations, we developed a CyTargetLinker tutorial presentation using Reveal.js. This tutorial presentation can be reused and adapted for specific teaching activities. Together with our tutorials for the Cytoscape desktop application and the automation example scripts, relevant documentation for users is provided to get familiar with the functionality of CyTargetLinker.

The generic nature of CyTargetLinker has been highlighted by renaming *RegINs* to *linksets*, and we now provide a variety of different linksets on our website. The XGMML structure of the linksets is simple, instructions how to create them from tab-delimited text files are available, and CyTargetLinker could therefore be used for non-biological networks as well (shown in use case 3).

## Conclusions

In this paper, we highlight the latest update of the CyTargetLinker app for Cytoscape and its new automation feature. The ability to programmatically execute the app’s functionality opens up the possibility to build complex workflows that are repeatable and reproducible. We also explored the broader applicability of the app besides the originally intended use for regulatory network extension. Due to the flexible design of the app and the linksets, we are now also showcasing other use cases, including non-biological networks.

## Data availability

Linksets, tutorials and link to source code (app and linkset creator) are available from the CyTargetlinker app website: https://cytargetlinker.github.io/.

## Software availability

1. **The app is available from the Cytoscape app store:** http://apps.cytoscape.org/apps/cytargetlinker.
2. **Link to source code:** https://github.com/CyTargetLinker/cytargetlinker.
3. **Archived source code at time of publication:** https://doi.org/10.5281/zenodo.3362389 ^20^.
4. **Tutorials including R code for use cases:** https://github.com/CyTargetLinker/cytargetlinker-automation.
5. **Software license:** https://www.apache.org/licenses/LICENSE-2.0.
