# Supplementary material for: CyTargetLinker app update: A flexible solution for network extension in Cytoscape
Source: F1000Res. 2019 Aug 13;7:ELIXIR-743. Originally published 2018 Jun 14. [Version 2] doi: 10.12688/f1000research.14613.2 (PMC6707396; doi:10.12688/f1000research.14613.2)
Supplement: Peer review history

# Peer review: Reviewer response for version 2

**DOI:** 10.5256/f1000research.22256.r52392

**Published:** 2019-08-22

**Authors:** John H. Morris

**Affiliations:** 1 Resource for Biocomputing, Visualization and Informatics, Department of Pharmaceutical Chemistry, University of California, San Francisco, San Francisco, CA, USA

I thank the authors for their careful attention to my comments, and particularly their efforts in improving the CyTargetLinker web cite. I believe they have addressed all of my concerns, or explained why making the requested changes would be onerous.

I confirm that I have read this submission and believe that I have an appropriate level of expertise to confirm that it is of an acceptable scientific standard.

# Peer review: Reviewer response for version 1

**DOI:** 10.5256/f1000research.15902.r40334

**Published:** 2018-12-05

**Authors:** John H. Morris

**Affiliations:** 1 Resource for Biocomputing, Visualization and Informatics, Department of Pharmaceutical Chemistry, University of California, San Francisco, San Francisco, CA, USA

The authors present an update to their successful CyTargetLinker app for Cytoscape.  This update extends the basic capabilities of CyTargetLinker by generalizing some concepts and adding automation functionality.  The article is clear and well-written and succinctly outlines the major point of the article and the features the article is going to focus on.  They present three use cases, which outline the automation capabilities nicely.

 I did have several issues that I believe should be addressed to support users trying to follow the instructions and to elucidate more clearly the functionality of the new automation features.

 First, the instructions point to a GitHub repository for the three use cases, however, I could only find two use cases in the repository.  This should absolutely be fixed before approval.

 Second, the authors state that both the ChEMBL and OMIM linksets are available on the CyTargetLinker website.  Unfortunately, only the ChEMBL linkset is provided.  The OMIM linkset **is** provided in the github repository, however, once the file is unzipped.  It wasn't clear to me why the authors chose to provide the data as a zip file in UseCase1, but just provide the unzipped directories in UseCase2.  I would suggest just to leave the files unzipped in UseCase1 in the same way you do for UseCase2.

 Third, to support general understanding of the automation, I would add the CyTargetLinker commands you actually execute to achieve the results.  For UseCase1, this would be:

 "cytargetlinker extend idAttribute=\"display name\" linkSetFiles=\"/tmp/cytargetlinker-tutorials/R/UseCase1/LinkSets/chembl_23_hsa_20180126.xgmml,/tmp/cytargetlinker-tutorials/R/UseCase1/LinkSets/rare-disease-gene-associations-hsa-20180411.xgmml\" network=current".  Obviously, the paths could be truncated, so something like:

 "cytargetlinker extend idAttribute=\"display name\" linkSetFiles=\" *path*/chembl_23_hsa_20180126.xgmml, *path*/rare-disease-gene-associations-hsa-20180411.xgmml\" network=current"

 This would make it much easier for app writers to utilize your command features without having to look through the R source code.

 And, as mentioned before, I couldn't find UseCase3.

 Finally, as a suggestion, it would be nice to have the functionality to create a linkset from a csv file as part of the app so the user wouldn't need to download and execute it separately.

 Overall, I'm enthusiastic about the app and the new capabilities, and would be happy to approve it after the above major changes are made.

I confirm that I have read this submission and believe that I have an appropriate level of expertise to state that I do not consider it to be of an acceptable scientific standard, for reasons outlined above.

# Response

**Published:** 2019-08-13

**Authors:** Martina Summer-Kutmon

Thank you for your comments. We completely restructured and updated the website. We also made sure the third use case script is available in the automation repository. 

 We provided the data for use case 1 as a zip file because the ChEMBL linkset is too large to be uploaded to Github. I now only zipped that specific linkset and gave instructions in the R script to first unzip that specific xgmml file. This makes it more consistent with the other use cases. 

 We added the general commands used to execute the extension for each of the use cases to the manuscript.

 Since we are using the linkset creator script to generate all linksets regularly, we opted for keeping it separate. Most users use the linksets provided or contact us to add a new one and only few generate their own. 

 We hope that we addressed all of your requests in version 2 and the new updated website.

# Peer review: Reviewer response for version 1

**DOI:** 10.5256/f1000research.15902.r35060

**Published:** 2018-06-28

**Authors:** Alex Gutteridge

**Affiliations:** 1 GlaxoSmithKline (GSK), Brentford, UK

The CyTargetLinker Cytoscape app presented here provides a useful mechanism for extending existing biological networks. The rationale for this functionality is well explained by the authors and the general utility is demonstrated by the citation of several previous studies using the tool and the variety of use cases presented.

 The API interface, which is the focus of this update, is a very useful addition which allows the scripting and hence reproducibility of this kind of analysis. The API is simple, but covers the fundamental use case of the app. I can only find partial documentation of the API method parameters however. They are named in Table 1, but I could not find more extensive documentation on the app website ( https://projects.bigcat.unimaas.nl/cytargetlinker/) which mostly refers to an earlier version of the app from what I can tell. The API methods should be documented somewhere more completely. This is why I have answered 'Partly' to 'Are sufficient details of the code, methods and analysis (if applicable) provided to allow replication of the software development and its use by others?'.

 The interpretation of the results and conclusions about the tool are straight forward. I have not tested the performance of the app outside the use cases the authors provide, but in those contexts performance is perfectly adequate. Perhaps the authors could comment on whether the app has performance limits on the size of network that can be annotated in this way and how that compares to Cytoscape's underlying limits.

 I tested the first use case script with Cytoscape 3.6.1, stringApp 1.1.1 and CyTargetLinker 4.0.1 (the latest versions at time of writing). This revealed a small API incompatibility. The script attempts to link on idAttribute 'display name', but no such attribute comes from this version of stringApp. Changing the idAttribute to 'name' allows the script to work. I can't find which version of stringApp the authors are using, but maybe it needs to be specified or the script updated. This is why I have marked 'Partly' to the question 'Are sufficient details of the code, methods and analysis (if applicable) provided to allow replication of the software development and its use by others?' Otherwise the use case reproduced fine.

 I have not tested the CyTargetLinker Java program, but it seems reasonably well documented and straight forward to run.

I confirm that I have read this submission and believe that I have an appropriate level of expertise to confirm that it is of an acceptable scientific standard.

# Response

**Published:** 2019-08-13

**Authors:** Martina Summer-Kutmon

Thank you for your comments. We completely restructured and updated the website and all tutorials and example scripts should now contain the relevant information to reproduce the results.
